# Intraperitoneal Rupture of Hepatic Hydatid Cyst Following Blunt Abdominal Trauma

**Published:** 2012-06-01

**Authors:** Anjan Kumar Dhua, Akshay Sharma, Yogesh Kumar Sarin

**Affiliations:** Department of Pediatric surgery, Maulana Azad Medical College, Delhi 110002, India.

**Keywords:** Hepatic hydatid cyst, Peritonitis, Trauma

## Abstract

Peritonitis due to rupture of liver hydatid cyst secondary to blunt abdominal trauma can present with fatal consequences. Timely diagnosis and appropriate surgical management can be life saving. We report a case of ruptured liver hydatid cyst in the peritoneal cavity following trauma and its successful operative management in a preadolescent previously asymptomatic boy. Importance of detailed physical examination and early diagnosis by using appropriate radiological investigations is highlighted.

## INTRODUCTION

Hepatic hydatid cysts can present in a myriad fashion. Acute presentation with rupture into the peritoneal cavity is a rare form of presentation with an incidence ranging from 1% to 8% [1, 2]. Intra-biliary rupture is another form of acute presentation and is more common than intraperitoneal rupture [3]. Following intraperitoneal rupture, presentation is usually acute with abdominal signs, such as guarding, and rebound tenderness with anaphylactic reactions occurring in 1% to 12.5% of cases, which at times could be life threatening [4, 5]. Herein, we report a preadolescent boy who presented with peritonitis following traumatic rupture of hydatid cyst of liver. The role of clinical examination and ultrasonography is highlighted for prompt diagnosis and successful management of this form of acute abdomen.

## CASE REPORT

An apparently healthy 11-year-old boy presented with acute pain in central abdomen of 5 hours duration. He gave history of a trivial blunt abdominal trauma while at play. Patient also complained of a bothersome itching all over his body especially over limbs and the trunk starting soon after the injury. At presentation, the patient was lying still in bed with pulse rate of 110/min and BP 110/60 mm Hg. There was no pallor. Lesions resembling utricaria were seen over thigh and trunk. Abdominal examination revealed generalized rebound tenderness.

Baseline investigations were normal except for leukocytosis (13200/ mm3). Roentgenograms of the chest and the abdomen were essentially normal. Ultrasonography revealed that liver was enlarged and there were 2 cystic lesions [6.4X4.6 cm and 8.1X 6.9 cm] in right lobe of liver with hypoechoic contents and floating echogenic membranes and peripheral calcifications. Lot of free fluid was also present.

Intravenous fluids were started with a bolus of Ringer lactate (20ml/Kg) followed by Dextrose 5% in normal saline (0.9%). Intravenous Hydrocortisone and Pheniramine maleate were adminstered along with antibiotic prophylaxis. The clinical picture with sudden generalized pain in abdomen with rashes, frank peritonitis and sonographic findings were suggestive of ruptured hepatic hydatid cyst. Patient was taken for exploratory laparotomy. The peritoneal cavity was filled with approximately 500 ml of bilious fluid which was drained out. Inspecting the liver surface showed extruded bile stained flaccid hydatid cyst (Fig. 1).

**Figure:**
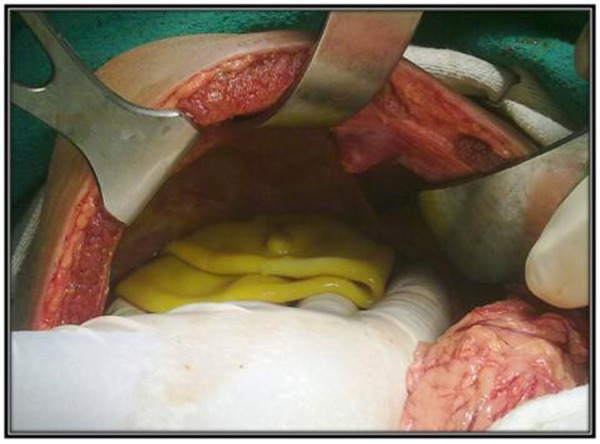
Figure 1: Extruded flaccid bile stained endocyst.

Another intact hydatid cyst was palpable in segment VI which was removed by partial pericystectomy. The entire peritoneal cavity was lavaged with hypertonic (3%) and normal saline. The bed of the first cyst was inspected for any bile leak. As there was no evidence of large cysto-biliary communication, the cavity was packed with omentum. A tube drain was placed in right sub-hepatic space. Postoperative course was uneventful. The drain was removed on the on 7th post operative day. albendazole (15 mg/kg/day) was started and plan was to continue it for 6 months (3 weekly courses and drug free period of one week with a watch on the liver enzymes and counts).

Four weeks later, the patient presented with upper abdominal fullness not associated with any other complaint. He was afebrile and hemodynamically stable. There was no icterus. On examination, there was distension of abdomen limited to the upper half of abdomen. There was no demonstrable free fluid and bowel sounds were normal. Laboratory values were: Hb-9.2 gms/dl, TLC-6900/mm3, serum bilirubin-0.8 mg%, ALT-17 U/ L, ALP 365 U/ L, AST-28 U/ L. Sonography revealed a large multiloculated cystic mass (15cmx13cmx18 cm) antero-superior to the liver. The intra-hepatic biliary radicals and the common bile duct were not dilated. There was no free fluid. CT scan was done to know further details. It showed 13cmx13cmx16 cm cystic lesion in the right lobe of liver with well defined septa of liver parenchyma within it (Fig. 2). Another cystic lesion was found in the left sub-hepatic space and lesser sac. Based on the findings it was diagnosed to be a “walled off” bile collection. A pig tail catheter was inserted percutaneously into the bilioma under sonographic guidance which was both diagnostic and therapeutic. It drained about 500 ml of greenish brown fluid overnight and culminated with disappearance of abdominal distension. The catheter was removed after 4 days when the effluent was negligible. Patient was discharged and on follow up 3 weeks later, found to be doing well. Currently patient is on albendazole therapy.

**Figure:**
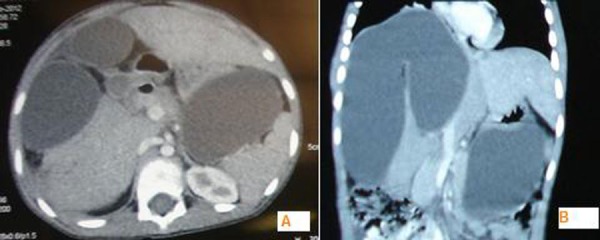
Figure 2: A-Axial section of CT scan showing cystic lesion in the right lobe of the liver with well defined septa of liver parenchyma within it. The lesion in lesser sac is also appreciated B- Same in coronal section.

## DISCUSSION

Ecchinococcus granulosus causes hydatid disease, most commonly in the liver. It is endemic in sheep farming areas such as South and Central America, Western Europe, the Middle East and some sub-Saharan countries. Humans are accidental intermediate hosts and in its life cycle, can be considered as a dead end. Presentation may be myriad ranging from asymptomatic to life threatening anaphylaxis. Rupture of a hydatid cyst after trauma is an uncommon presentation especially in a non endemic area. Few reports of intraperitoneal rupture have been reported from the Indian subcontinent. Ray et al [6] reported spontaneous intraperitoneal and intra-biliary rupture of hepatic hydatid cyst in a 34 year old man. Ahuja et al [7] reported a 10–year-old child with hepatic hydatid cyst rupturing into sub-diaphragmatic space and pericardial cavity. The presentation may be quite dramatic because of the gross spillage of the contents that not only cause chemical peritonitis, but sometimes can also cause anaphylaxis. If there is bile leak, it further exacerbates the inflammatory process in the peritoneum.

Preoperative diagnosis can be missed especially in a patient like ours who did not have any suggestive history except for the utricaria like lesions. Both ultrasonography and computerized tomography are highly sensitive in demonstrating the cyst rupture [4]. However the combination of clinical examination and ultrasonography findings made the diagnosis quite obvious which was further confirmed on laparotomy. Serology is used to detect specific serum antibodies or circulating antigens by a variety of immunodiagnostic methods. The most commonly used technique is enzyme linked immunosorbent assay (ELISA) for detection of echinococcal antibodies (IgG) in the serum. A false positive value can occur in a normal person especially in endemic area and similar results can also occur in patients with other parasitic infestations [8].

Treatment has to be done expeditiously to prevent spreading of chemical peritonitis and to prevent disseminated peritoneal echinococcosis. Surgical therapy is no doubt the mainstay but the exact approach, radical or conservative is still not defined. In an emergency setting, the conservative methods have shown to yield satisfactory results [9]. Various obliterative techniques (omentoplasty, capitonage, and intraflexion) have been described in the literature [3]. Some authors have favoured pericystectomy and liver resections as preferred treatment for hydatid disease [10]. Pericystectomy are usually performed when the location of the cyst is away from major biliary or vascular structures. Some form of liver resection is possible in an emergency setting only if the cyst is peripheral and pedunculated. We performed partial pericystectomy with drainage. Akcan et al in a series of 372 patients with hydatid cysts did partial pericystectomy with drainage in 70% of patients as their preferred surgical technique [3]. The authors found this technique superior to others because it was simple, applicable to almost all cysts irrespective of its location and a shorter operation time.

After removal of the entire cyst and its contents, the peritoneal cavity should be liberally lavaged with scolicidal agents and normal saline. Various agents and hypertonic saline have been described in the literature for the purpose [11]. Gargouri et al used 3%-5% saline with equal efficacy but stressed on the importance of contact time for effectiveness [12]. The cystobilious communication should be dealt with depending on the size of the fistulae. For large communications T-tube drainage [13], choledochoduodenostomy or sphincterotomy has been described. Endoscopic biliary stenting has also been reported to be of value in treatment for biliary fistulas complicating liver hydatid cysts [14]. In our case, the delayed appearance of bilary collection was most likely because of minor cystobiliary communications which somehow remained patent even after 4 weeks of initial surgery. An intra-biliary rupture was ruled out because patient did not have jaundice or features of cholangitis. The sonography and CT scan were also not suggestive of intra-biliary rupture.

To prevent recurrences, albendazole therapy is mandatory. It should be started immediately and given for a prolonged period of time [15]. Follow up with 6 monthly imaging is prudent to detect recurrences. If these basic principles are followed the recurrences in these cases are not as high as once thought. The purpose of this report is to sensitize the surgeons dealing with emergency and trauma patients to consider rupture of Hydatid cyst in the differential diagnosis of acute post traumatic peritonitis and to reemphasize that a carefully obtained history and physical examination aided by radiological investigation can help diagnose them without undue delay. 

## Footnotes

**Source of Support:** Nil

**Conflict of Interest:** None declared
